# Physiological profiles associated with ceasing growth of unfertilized eggs produced by unmated queens in the subterranean termite *Reticulitermes chinensis*

**DOI:** 10.1242/bio.017319

**Published:** 2016-05-23

**Authors:** Ganghua Li, Long Liu, Pengdong Sun, Yao Wu, Chaoliang Lei, Xiongwen Chen, Qiuying Huang

**Affiliations:** 1 College of Plant Science and Technology, Huazhong Agricultural University, Wuhan, Hubei 430070, China; 2 College of Life Science, Hubei Normal University, Huangshi, Hubei 435002, China

**Keywords:** Termites, Asexual queen succession, Ovarian development, Embryonic growth, Physiological indices

## Abstract

In *Reticulitermes chinensis*, a close relative of *R.* *speratus* with asexual queen succession, unfertilized eggs can be produced but do not hatch as larvae. To explain this phenomenon, we analyzed the physiological differences between unfertilized eggs/unmated queens and fertilized eggs/mated queens. Fertilized eggs had significantly lower quantities of five amino acids (Cys, Met, Ile, Leu and Tyr), Ca, protein and cholesterol during development. The higher levels of four trace elements (Na, K, Zn and Fe) in fertilized eggs and their lower levels in mated queens indicated that mated queens might transfer these trace elements to fertilized eggs to aid development. The higher levels of Mn, triglycerides and serotonin in mated queens and higher levels of Mn and glucose in fertilized eggs suggested that these substances are very important for normal ovarian and embryonic growth. The different expression of three reproductive genes (*vtg 1*, *rab 11* and *JHE 1*) suggested that they might be involved in the regulation of ovarian and embryonic growth. Overall, changes in these physiological indices may substantially affect ovarian and embryonic growth and inhibit development of unfertilized eggs in *R. chinensis*.

## Abstract (precis)

**Summary:** Changes in amino acids, trace elements, nutrient content, serotonin and reproductive genes may substantially affect ovarian and embryonic growth and inhibit development of unfertilized eggs in *Reticulitermes chinensis*.

## INTRODUCTION

Recently, asexual queen succession (AQS) has been described in three species of lower termites [*Reticulitermes speratus* (Matsuura et al., 2009), *R.* *virginicus* (Vargo et al., 2012) and *R.* *lucifugus* (Luchetti et al., 2013)] and two species of higher termites [*Embiratermes neotenicus* (Fougeyrollas et al., 2015) and *Cavitermes tuberosus* (Roisin et al., 2014)]. In AQS species of termites, workers, soldiers and alates are sexually produced but neotenic queens arise through thelytokous parthenogenesis (Matsuura et al., 2009). This AQS system enables the primary queen to maintain her full genetic contribution to the next generation while avoiding any loss of genetic diversity from inbreeding (Matsuura, 2011). Most of the work conducted on the AQS system in termites has focused on the discovery of new AQS species and the mechanism of facultative parthenogenesis (Fougeyrollas et al., 2015; Kawatsu and Matsuura, 2013; Luchetti et al., 2013; Roisin et al., 2014; Vargo et al., 2012; Yashiro and Matsuura, 2014). Some termite species that are evolutionally related to AQS species of termites have been demonstrated to exhibit neither AQS nor parthenogenesis (Kawatsu and Matsuura, 2013; Luchetti et al., 2013); however little is known about why these termite species have no AQS and specifically why unfertilized eggs produced by unmated queens of these species are unable to hatch.

The ovarian and embryonic development of insects are both complex physiological processes that are influenced by many physiological factors, including nutrients (Bodnaryk and Morrison, 1966; Judd et al., 2010), trace elements (Chaudhury et al., 2000; Hammack, 1999), hormones (Hagedorn et al., 1975; Lagueux et al., 1977) and reproductive genes (e.g. vitellogenin genes) (Ishitani and Maekawa, 2010; Maekawa et al., 2010). For example, wasp eggs require substantial quantities of proteins and lipids to provide the materials and energy for embryonic growth (Judd et al., 2010). Adult female house flies require an adequate protein diet to initiate and sustain normal ovarian development (Bodnaryk and Morrison, 1966). In adult mosquitoes (Hagedorn et al., 1975) and adult females of *Locusta migratoria* (Lagueux et al., 1977), ecdysone plays an important role in stimulating egg development and vitellogenin synthesis. Metal ions participate in the process of enzyme activation and in trigger and control mechanisms (Chaudhury et al., 2000), for example, potassium (K) was reported to be a key ion for protein-dependent egg maturation in three insects, *Phormia regina*, *Sarcophaga bullata*, and *Cochliomyia hominivorax* (Chaudhury et al., 2000; Hammack, 1999). Previous studies on ovarian development in termites have primarily focused on changes in ovarian morphology, ovarian dynamics, factors affecting ovarian development, juvenile hormone (JH) titres and the expression of vitellogenin genes at different queen stages in termites (Watson, 1972; Greenberg et al., 1978; Brent and Traniello, 2001; Brent et al., 2007; Liebig et al., 2009; Ishitani and Maekawa, 2010; Maekawa et al., 2010). With regard to embryonic growth in termites, differences in embryonic development, size, survival rate and the length of the hatching period between unfertilized and fertilized eggs have been investigated in termites (Gillott, 2005; Matsuura and Kobayashi, 2007); however the physiological differences between unfertilized eggs/unmated queens and fertilized eggs/mated queens, such as the content of amino acids, nutrients, trace elements, hormones, neurotransmitters and the expression of reproductive genes, have not been investigated.

The subterranean termite *R. chinensis*, which is widely distributed in China, causes serious damage to buildings and forests and results in major economic losses (Liu et al., 2015). In the present study, we found that unfertilized eggs can be produced by unmated queens of *R. chinensis*, but they do not hatch under laboratory and simulated field conditions, suggesting that *R. chinensis* exhibits neither AQS nor parthenogenesis. To explore why unfertilized eggs of *R*. *chinensis* are unable to hatch, we conducted a comprehensive analysis of physiological differences in ovarian and embryonic growth between unfertilized eggs/unmated queens and fertilized eggs/mated queens. We found that unfertilized eggs ceased embryonic growth and had significant differences in morphological characters, size and micropyle (sperm gate) number compared with fertilized eggs in the final stage (stage V) of development. Moreover, there were significant differences in the levels of 11 amino acids, six trace elements, four nutrients, serotonin, and the expression of three reproductive genes (*vtg 1*, *rab 11* and *JHE 1*) between unfertilized eggs/unmated queens and fertilized eggs/mated queens, suggesting that the absence of hatching of unfertilized eggs produced by unmated queens is associated with the above changes in physiological indices in *R. chinensis*. Our physiological findings contribute to an understanding of why the subterranean termite *R. chinensis* exhibits neither AQS nor parthenogenesis even though it is a close relative of *R. speratus*, which does exhibit AQS (Austin et al., 2004; Matsuura et al., 2009).

## RESULTS

### Formation of female-female colonies and female-male colonies under laboratory and simulated field conditions

**Fig. 1.**
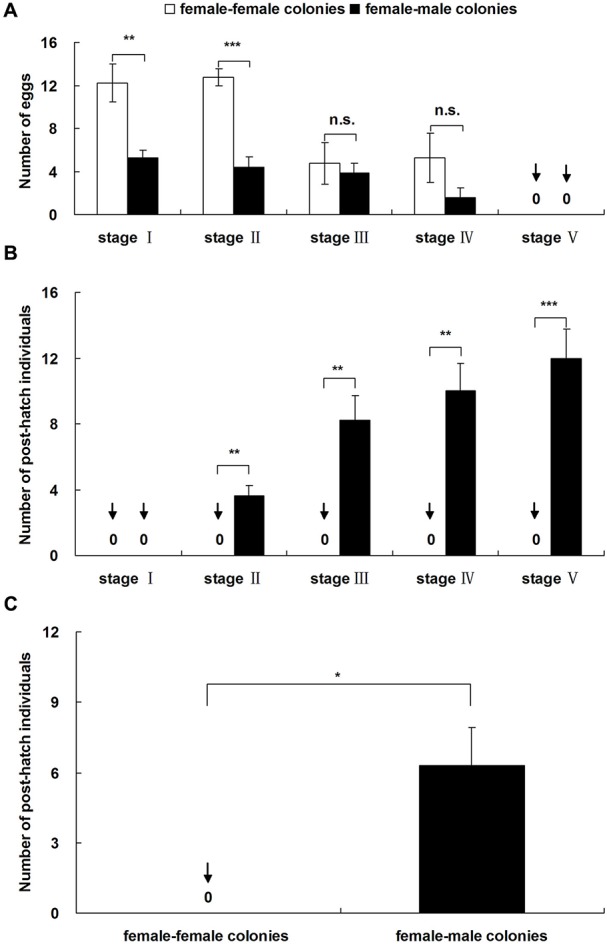
**Comparison of offspring number between female-female and female-male colonies.** (A) Eggs and (B) post-hatch individuals under laboratory conditions. (C) Post-hatch individuals under simulated field conditions. Data represented as mean±s.e.m. Asterisks denote significant differences by paired *t*-test; A and B: *n*=8, C: *n*=16; n.s., not significant; *P<0.05, **P<0.01, ***P<0.001.

The number of unfertilized eggs in female-female (FF) colonies was significantly higher than the number of fertilized eggs in female-male (FM) colonies at stages I and II (Fig. 1A; stage I: *P*=0.001; stage II: *P*<0.001). The numbers of both unfertilized and fertilized eggs decreased during stages III and IV, and no significant differences were found (Fig. 1A; stage III: *P*=0.643; stage IV: *P*=0.214). At stage V, no newly produced eggs were found in either the FF or FM colonies.

No post-hatch individuals were observed in any FF colony at all five developmental stages (Fig. 1B). Post-hatch individuals (larvae, workers, pre-soldiers or soldiers) were found after stage II in the FM colonies (Fig. 1B). Larvae (1-5 individuals per colony) were found at stage II in all eight FM colonies (8/8 colonies: 100%). At stage III, all the eight FM colonies (100%) contained workers (2-8 individuals per colony). Two pre-soldiers were found in two of the FM colonies at stage IV (2/8 colonies: 25%). Five soldiers were found in five colonies during stage V (5/8 colonies: 62.5%). The number of post-hatch individuals in the FM colonies was significantly higher than in FF colonies for stages II to V (Fig. 1B; stages II-V: *P*<0.01).

In simulated field conditions, no post-hatch individuals were found in any of the FF colonies 5.5 months after colony formation; however post-hatch individuals (average 6.29 individuals per colony) were found 5.5 months after colony formation in all the FM colonies (Fig. 1C). The number of post-hatch individuals in the FM colonies was significantly higher than in the FF colonies (Fig. 1C; *P*=0.024).

### Morphological observation of eggs at the five developmental stages

**Fig. 2.**
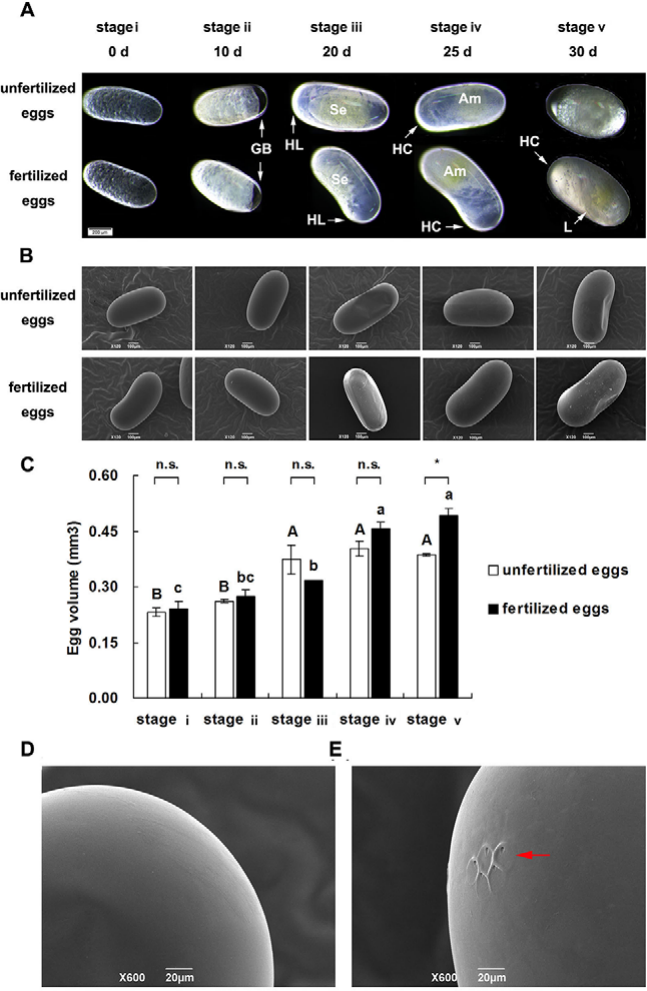
**Differences in morphological characters, size and micropyle number between unfertilized and fertilized eggs.** (A) Egg morphology under stereoscope. GB, germ band; HL, head lobe; HC, head capsule; Se, serosa; Am, amnion; L, leg. (B) Egg morphology of fertilized and unfertilized eggs under SEM. (C) Differences in egg size between unfertilized and fertilized eggs at five developmental stages under SEM. Data represented as mean±s.e.m. *n*=3; n.s., not significant, *P<0.05. (D,E) Unfertilized eggs without micropyles (D) and fertilized eggs with micropyles (E, red arrow) under SEM.

The embryos of fertilized eggs developed normally and exhibited visible differences among the five developmental stages (from stage i to stage v; Fig. 2A). In unfertilized eggs, 75% (60/80) embryos ceased development at stage ii, and 3.33% (2/60) and 5% (2/40) of the embryos developed to stage iii or stage iv respectively, but none of the embryos developed to stage v, unlike the fertilized eggs (Fig. 2A). There were no significant differences in morphological characters and size between unfertilized eggs and fertilized eggs prior to stage iv (Fig. 2A,B); however the unfertilized eggs gradually shrank and spoiled, and they were significantly smaller than fertilized eggs at stage v (Fig. 2A,C; *P*=0.008). Micropyles were located on the posterior end of the eggs. Almost all the unfertilized eggs (14/15) had no micropyles, but all fertilized eggs had micropyles (Fig. 2D).

### Amino acids

**Fig. 3.**
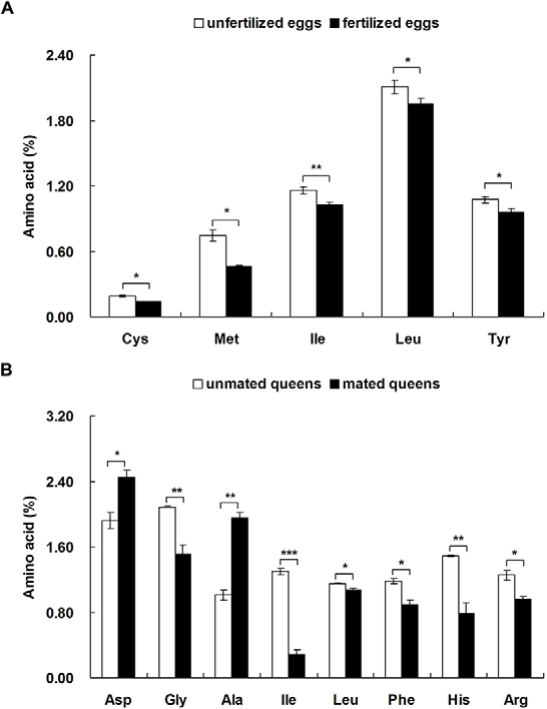
**Differences in the levels of 11 amino acids between unfertilized eggs/unmated queens and fertilized eggs/mated queens.** Data represented as mean±s.e.m. Asterisks denote significant differences by paired *t*-test; *n*=4; *P<0.05; **P<0.01; ***P<0.001.

The levels of five amino acids (Cys, Met, Ile, Leu and Tyr) in unfertilized eggs from FF colonies were significantly higher than in fertilized eggs from FM colonies (Fig. 3A; Table S1; Cys: *t*=−8.000, d.f.=2, *P*=0.015; Met: *t*=−6.424, d.f.=2, *P*=0.023; Ile: *t*=−10.250, d.f.=2, *P*=0.009; Leu: *t*=−5.277, d.f.=2, *P*=0.034; Tyr: *t*=−9.430, d.f.=2, *P*=0.011). The levels of two amino acids (Asp and Ala) in unmated queens were significantly lower than in mated queens (Fig. 3B; Asp: *t*=4.014, d.f.=2, *P*=0.016; Ala: *t*=10.123, d.f.=2, *P*=0.001), but the levels of six amino acids (Gly, Ile, Leu, Phe, His and Arg) in unmated queens were significantly higher than in mated queens (Fig. 3B; Gly: *t*=−5.354, d.f.=2, *P*=0.006; Ile: *t*=−15.296, d.f.=2, *P*<0.001; Leu: *t*=−3.101, d.f.=2, *P*=0.036; Phe: *t*=−4.177, d.f.=2, *P*=0.014; His: *t*=−5.494, d.f.=2, *P*=0.005; Arg: *t*=−5.087, d.f.=2, *P*=0.007).

### Trace elements

**Fig. 4.**
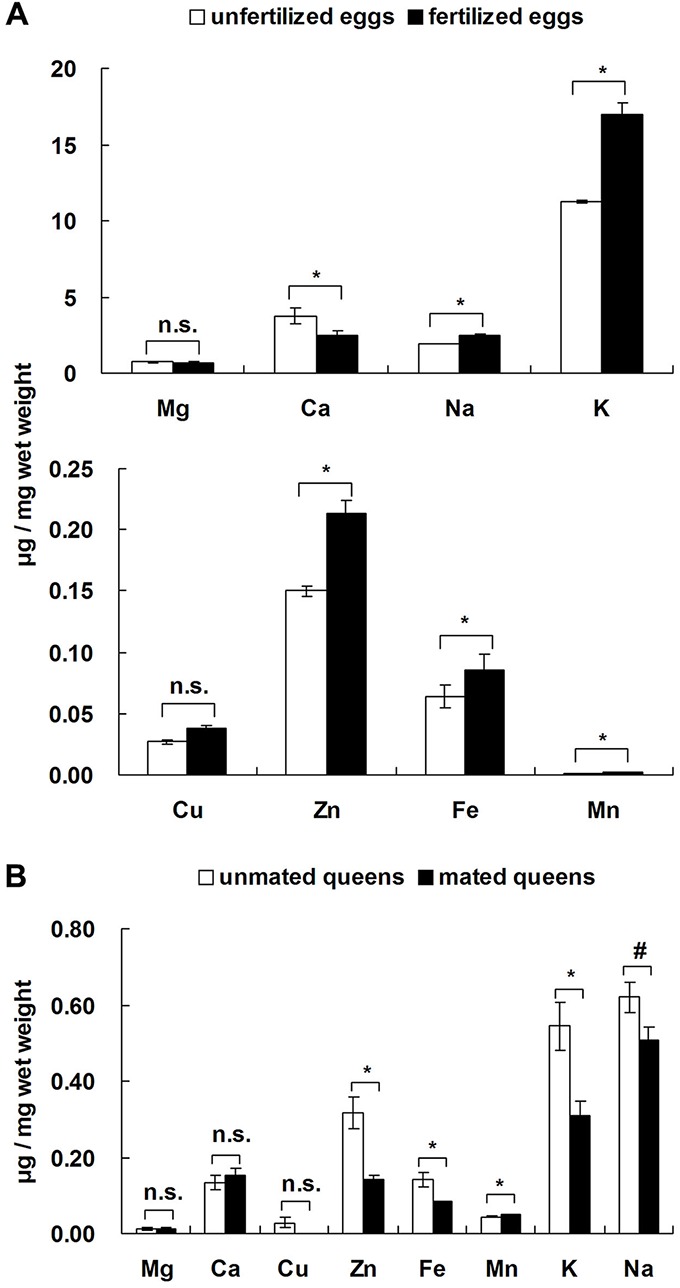
**Differences in the levels of eight trace elements between unfertilized eggs/unmated queens and fertilized eggs/mated queens.** Data represented as mean±s.e.m. Asterisks denote significant differences by paired *t*-test; *n*=4; n.s. not significant; ^#^0.1<*P*<0.05; *P<0.05.

The Ca level in unfertilized eggs from FF colonies was significantly higher than in fertilized eggs from FM colonies (Fig. 4A; *t*=−4.923, d.f.=2, *P*=0.039), but the levels of five trace elements (Na, K, Zn, Fe and Mn) in unfertilized eggs were significantly lower than in fertilized eggs (Fig. 4A; Na: *t*=26.179, d.f.=2, *P*=0.001; K: *t*=7.703, d.f.=2, *P*=0.016; Zn: *t*=4.436, d.f.=2, *P*=0.047; Fe: *t*=5.079, d.f.=2, *P*=0.037; Mn: *t*=5.038, d.f.=2, *P*=0.037). The levels of four trace elements (Zn, Fe, K and Na) in unmated queens were significantly or marginally significantly higher than in mated queens (Fig. 4B; Zn: *t*=4.080, d.f.=2, *P*=0.015; Fe: *t*=3.075, d.f.=2, *P*=0.037; K: *t*=3.203, d.f.=2, *P*=0.033; Na: *t*=2.163, d.f.=2, *P*=0.097). However, the Mn level in unmated queens was significantly lower than in mated queens (Fig. 4B; *t*=−3.878, d.f.=2, *P*=0.018).

### Nutrient content

**Fig. 5.**
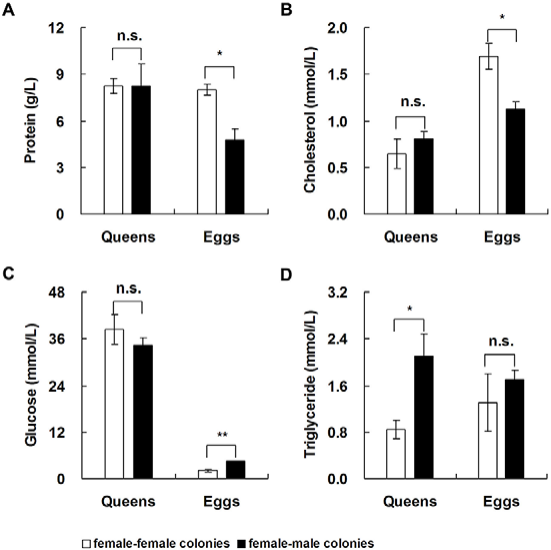
**Differences in the content of four nutrients between unfertilized eggs/unmated queens and fertilized eggs/mated queens.** Data represented as mean±s.e.m. Asterisks denote significant differences by paired *t*-test; *n*=4; n.s. not significant; *P<0.05; **P<0.01.

The levels of proteins and cholesterol in unfertilized eggs from FF colonies were significantly higher than in fertilized eggs from FM colonies (Fig. 5A, protein: *t*=4.038, d.f.=4, *P*=0.016; Fig. 5B, cholesterol: *t*=3.500, d.f.=4, *P*=0.025), but the glucose level of unfertilized eggs was significantly lower than in fertilized eggs (Fig. 5C; *t*=−6.124, d.f.=4, *P*=0.004). There were no significant differences in the triglyceride level between unfertilized and fertilized eggs, but the triglyceride level of unmated queens was significantly lower than in mated queens (Fig. 5D; *t*=−2.906, d.f.=4, *P*=0.044).

### Hormones and neurotransmitters

**Fig. 6.**
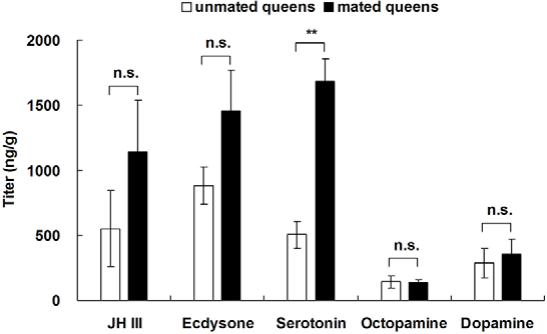
**Differences in the levels of two hormones and three neurotransmitters between unmated and mated queens.** Data represented as mean±s.e.m. Asterisks denote significant differences by paired *t*-test; *n*=4; n.s. not significant; **P<0.01.

Levels of the neurotransmitter serotonin in unmated queens from FF colonies were significantly lower than in mated queens from FM colonies (Fig. 6; *t*=−5.867, d.f.=4, *P*=0.004), but there were no significant differences in the levels of two hormones (JH III and ecdysone) or other two neurotransmitters (octopamine and dopamine) between unmated and mated queens (Fig. 6).

### Reproductive genes

**Fig. 7.**
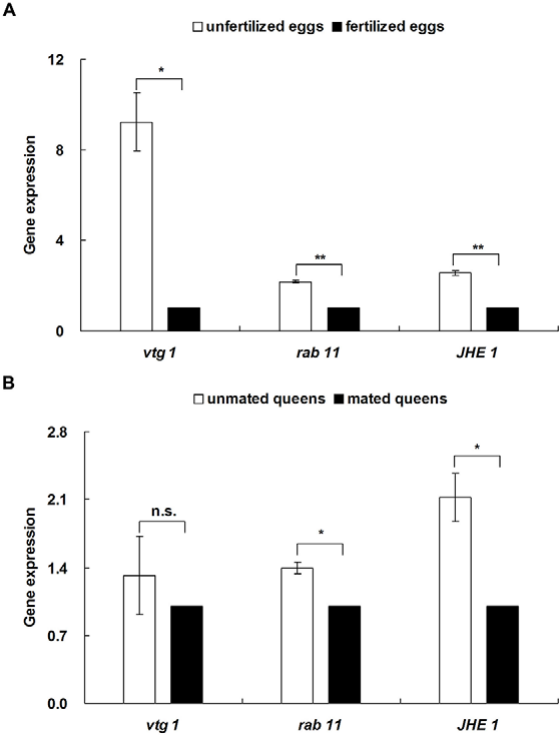
**Differences in the expression of three reproductive genes between unfertilized eggs/unmated queens and fertilized eggs/mated queens.** Data represented as mean±s.e.m. Asterisks denote significant differences by paired *t*-test; *n*=4; n.s. not significant; *P<0.05; **P<0.01.

The expression of three reproductive genes in unfertilized eggs from FF colonies was significantly higher than in fertilized eggs from FM colonies (Fig. 7A; *vtg 1*: *t*=6.319, d.f.=2, *P*=0.024; *rab 11*: *t*=17.528, d.f.=2, *P*=0.003; *JHE 1*: *t*=15.400, d.f.=2, *P*=0.004). Moreover, the expression levels of two reproductive genes in unmated queens were significantly higher than in mated queens (Fig. 7B; *rab 11*: *t*=6.614, d.f.=2, *P*=0.022; *JHE 1*: *t*=4.510, d.f.=2, *P*=0.046).

## DISCUSSION

In this study, none of the unfertilized eggs produced by unmated queens from colonies of *R. chinensis* hatched under laboratory or simulated field conditions, suggesting that parthenogenesis does not occur in *R. chinensis*, which is consistent with the absence of AQS in *R. chinensis* inferences from the presence of heterozygote genotypes in neotenic reproductives of *R*. *chinensis* (Huang et al., 2013) and the lack of a female-biased sex ratio in the flying alates of *R. chinensis* (Li et al., 2015). Our results indicated that the embryos of all unfertilized eggs ceased growth prior to stage V, potentially due to a lack or reduction of certain important physiological substances. In addition, almost all of the unfertilized eggs (14/15) lacked micropyles but all of the fertilized eggs had micropyles in *R. chinensis*; parthenogenetic eggs of *R. speratus* also lack micropyles (Yashiro and Matsuura, 2014).

Amino acids play an important role in ovarian and embryonic development in animals (Osako et al., 2007; Sato et al., 2009). We found that the levels of five amino acids (Ile, Leu, Cys, Met and Tyr) in unfertilized eggs were significantly higher than in fertilized eggs. A possible explanation for this result is that the sustained embryonic growth of fertilized eggs requires considerable quantities of these five amino acids to aid development. These data are consistent with observations in mice that embryonic growth requires substantial levels of Cys (de Matos et al., 2003). Mated queens had significantly lower levels of six amino acids (Gly, Ile, Leu, Phe, His and Arg), suggesting that mated queens must consume large quantities of these amino acids to complete ovarian development after mating in *R. chinensis*. Compared with unmated queens, the significantly higher levels of two amino acids (Asp and Ala) in mated queens may be derived from male reproductives via mating or trophallaxis.

Several ions, including Ca, K, Na, Cu and Zn, are important for growth, ovarian development, fecundity, and embryonic growth in insects (Judd et al., 2010; McFarlane, 1991). The levels of four trace elements (Na, K, Zn and Fe) in fertilized eggs were significantly higher than in unfertilized eggs, but their levels in mated queens were significantly lower than in unmated queens. A possible explanation for this phenomenon is that mated queens transfer their Na, K, Zn and Fe to fertilized eggs. These four trace elements may be necessary during embryonic growth and hatch as larvae. These data are consistent with a previous study that found that a decrease in Zn and/or Fe levels resulted in the cessation of growth in human embryos (Bai et al., 2014). The significantly higher levels of Mn in fertilized eggs and mated queens indicated that Mn plays an important role in ovarian and embryonic development during sexual reproduction. These data are consistent with the observation that a lack of Mn decreases the hatchability of eggs in chickens (Leach and Gross, 1983). In addition, the Ca level in fertilized eggs was significantly lower than in unfertilized eggs, suggesting that fertilized eggs require substantial levels of Ca to sustain embryonic growth during development. Similarly, normal embryonic development of eggs in turtles, crocodiles and birds consumes considerable quantities of Ca and obtains a portion of the Ca from the eggshell (Dunn and Boone, 1977).

Macronutrients (protein, glycogen, and lipid) play a crucial role in embryonic growth (Diss et al., 1996; Sloggett and Lorenz, 2008) in insects. In the present study, the levels of proteins and cholesterol in fertilized eggs were significantly lower than in unfertilized eggs, suggesting that the sustained embryonic growth and development of fertilized eggs requires more protein and cholesterol than in unfertilized eggs (Diss et al., 1996; Moran, 2007). However, the glucose level in fertilized eggs was significantly higher than in unfertilized eggs, implying that glucose may be a primary energy source in the late stages of embryonic growth (Ding et al., 2008). Mated queens had significantly higher levels of triglycerides than unmated queens, suggesting that triglycerides are an important energy source during ovarian development after mating in *R. chinensis*. This result is consistent with the rapid increase in triglycerides observed during ovarian development in the mated female adults of *Marsupenaeus japonicus* (Cavalli et al., 2001). A portion of the triglycerides in mated queens may be derived from males via mating or trophallaxis (Matsuura and Nishida, 2001; Shellman-Reeve, 1990).

Little is known about which genes are involved in the regulation of ovarian and embryonic growth in termites (Ishitani and Maekawa, 2010; Maekawa et al., 2010). We found that the expression levels of two reproductive genes (*rab 11* and *JHE 1*) in unfertilized eggs and unmated queens were significantly higher than in fertilized eggs and mated queens. A previous study demonstrated that Rab 11 is a small GTP binding protein involved in vesicular trafficking and plays a crucial role in the fertility of *Drosophila* (Tiwari et al., 2008). Juvenile hormone esterase (JHE), a member of the carboxylesterase family, contributes to the rapid decline in JH in most insects. JH is well known to play an important role in the reproductive competence and caste polyphenism of termites (Cornette et al., 2008; Elliott and Stay, 2008; Zhou et al., 2007). Thus, we predicted that these two reproductive genes (*rab 11* and *JHE 1*) might play a role in the ovarian and embryonic development of *R. chinensis*. In this study, we did not find significant differences in two other hormones (JH III and ecdysone) between mated queens and unmated queens, but the serotonin level of mated queens was significantly higher than in unmated queens. As a neurotransmitter, serotonin is correlated with reproductive behavior in mice (Liu et al., 2011; Zhang et al., 2013), suggesting that it might also play an important role in the sexual reproduction of *R. chinensis*. Vitellogenin (Vtg) is the dominant egg yolk protein in insects (Sappington and Raikhel, 1998). The expression of the gene *vtg 1* in fertilized eggs was significantly lower than in unfertilized eggs in this study, suggesting that the gene *vtg 1* was associated with embryonic development in fertilized eggs.

In conclusion, unfertilized eggs can be produced by unmated queens of *R. chinensis* but do not hatch under laboratory and simulated field conditions, suggesting that *R. chinensis* exhibits neither AQS nor parthenogenesis. Through physiological analyses, we found that unfertilized eggs ceased embryonic growth and had significant differences in morphological characters, size and micropyle number compared with fertilized eggs in the final stage of development. Moreover, fertilized eggs had significantly lower quantities of five amino acids (Cys, Met, Ile, Leu and Tyr), Ca, protein, and cholesterol during embryonic growth and development. Fertilized eggs may obtain a portion of four trace elements (Na, K, Zn and Fe) from mated queens, which facilitate embryonic growth during development. The significantly higher levels of Mn and glucose in fertilized eggs than unfertilized eggs suggest that these substances play an important role in the completion of embryonic growth and development. The significantly higher levels of triglycerides and serotonin in mated queens than in unmated queens imply that they are very important for ovarian development during sexual reproduction. The greater expression of three genes (*vtg 1*, *rab 11* and *JHE 1*) in unfertilized eggs and two genes (*rab 11* and *JHE 1*) in unmated queens suggests that these reproductive genes may be involved in the regulation of ovarian and embryonic growth in *R. chinensis*. Overall, the differences in these physiological indices may substantially affect ovarian and embryonic growth and inhibit development of unfertilized eggs in *R. chinensis*. Our physiological findings contribute to an understanding of why the subterranean termite *R. chinensis* exhibits neither AQS nor parthenogenesis even though it is a close relative of *R. speratus*, which exhibits AQS.

## MATERIALS AND METHODS

### Formation of female-female colonies and female-male colonies under laboratory conditions

The alates of *R. chinensis* used in this study were collected together with nest wood just prior to the swarming season. In April 2013, four mature colonies were collected from Nanwang Hill (A), Yujia Hill (B), and Shizi Hill (C and D), in Wuhan City, China. The termites were reared in an open plastic container (670×480×410 mm^3^) covered by nylon mesh in a dark room with a temperature of 16°C for 14 days and then moved to a room with a temperature of 30°C to help the alates fly (Matsuura et al., 2002; Matsuura and Nishida, 2001). According to the method of Roonwal (1975), we separated male imagoes from female imagoes. The same-sex imagoes were then maintained together in Petri dishes containing moist filter paper until they shed their wings. All FF colonies were constructed using the following combinations: F_A_F_A_, F_B_F_B_, F_C_F_C_ and F_D_F_D_. All FM colonies were constructed using the following combinations: F_A_M_B_, F_B_M_C_, F_C_M_D_ and F_D_M_A_. Each founder unit was replicated eight times. The paired founders were placed in 15.5 ml wells of 6-well plates containing moist rotten pine sawdust and vermiculite and were maintained at 25°C in constant darkness. The colonies were then sampled at months 0.5, 1.5, 2.5, 3.5, and 7.5 (stages I-V, respectively). For quantification of offspring (eggs, larvae, pre-soldiers, nymphs, workers and soldiers), the samples were placed in Petri dishes (9 cm diameter) containing moist filter paper after extracting the primary reproductives and their offspring. Offspring were quantified according to larval stages and mature castes, which was determined by the number of antennal segments and head width (Liu et al., 2001). None of the hills where samples were collected are privately-owned or protected in any way, and *R. chinensis* is not endangered or protected. Thus, no specific permissions were required to access and sample at these locations.

### Formation of female-female colonies and female-male colonies under simulated field conditions

In April 2012, three mature colonies were collected from Nanwang Hill (E), Yujia Hill (F), and Shizi Hill (G), in Wuhan City, China. We used the same method as described above for the laboratory conditions to help alates fly. Alates were randomly chosen from each colony and assigned to construct FF colonies or FM colonies. According to the method of Matsuura et al. (2004), FF colonies were constructed using the following combinations: F_E_F_E_, F_E_F_F_, F_F_F_F_, F_E_F_G_, F_F_F_G_ and F_G_F_G_; FM colonies were constructed using the following combinations: F_E_M_E_, F_E_M_F_, F_E_M_G_, F_F_M_E_, F_F_M_F_, F_F_M_G_, F_G_M_E_, F_G_M_F_ and F_G_M_G_. There were ten replicates for each combination. Each founder unit was placed in a fixed nest tube with a piece of rotten pine wood (11 cm diameter, 10 cm high, open at both ends, partially buried in the ground, with 3-4 cm between the ground and the bottom of the pipe). Each nest tube was covered by a small plastic bucket. The number of offspring in each colony was observed at 5.5 months after colony formation.

### Morphological observation of eggs at the five developmental stages

We used the same parent colonies and method as described above for the laboratory conditions to build founder combinations. All FF colonies were constructed using the following combinations: F_A_F_A_, F_B_F_B_, F_C_F_C_ and F_D_F_D_. All FM colonies were constructed using the following combinations: F_A_M_B_, F_B_M_C_, F_C_M_D_ and F_D_M_A_. Each founder unit was replicated five times. The paired founders were kept in a 50×70×2 mm^3^ glass cell (Matsuura et al., 2002). A mixed sterile vermiculite block was placed in the upper four-sevenths of the cell, and a 10 mm diameter hole with a 1.5 mm opening was cut out. A piece of balsa wood (5×5×1.5 mm^3^) was placed in the hole. The cell was covered with a 60×80 mm^2^ glass plate and was maintained at 25°C in constant darkness.

We observed the production of the first brood and collected eggs from the glass cells daily. Eggs were placed in a 1.5 ml microtube with 1 ml distilled water and vortexed for 30 s. They were then washed three times, placed on a 1.5% agar plate (90 mm) containing 200 ppm tetracycline under sterile conditions, and maintained at 25°C in the dark (Matsuura and Kobayashi, 2007). Eggs were sampled at five developmental stages: 0 day, 10 days, 20 days, 25 days, and 30 days after oviposition (stages i-v, respectively) (Matsuura and Kobayashi, 2007). The embryonic growth of the eggs was observed under a stereomicroscope with a transmitted light base (Olympus SZX16), photographed and measured using a digital imaging system (Matsuura and Kobayashi, 2007). In addition, we counted the number of micropyles on fertilized and unfertilized eggs of *R. chinensis* using a scanning electron microscope (SEM) (JSM-6390LV; NTC, Japan) (Yashiro and Matsuura, 2014). The sizes of 15 eggs randomly selected from each of the five developmental stages (three eggs per stage) were measured using the SEM. The length (a) and diameter (b) of each egg were measured using JEOL SMile View software for image processing. Approximate egg volumes were calculated using the formula *V*=4πab^2^/3 (Matsuura and Kobayashi, 2007).

### Amino acids

Unmated queens and unfertilized eggs from FF colonies and mated queens and fertilized eggs from FM colonies were sampled (queens were synchronously sampled 40 days after colony formation, and eggs were synchronously sampled 25 days after oviposition; the same below) for the analysis of amino acid composition and were hydrolysed with 6 M HCI under vacuum at 110°C for 24 h. After 24 h, the samples were dried in a vacuum chamber with a NaOH trap. The dried samples were solubilized in pH 2.2 citrate buffer and filtered through a cellulose acetate membrane (0.22 mm). A portion of the filtrate was injected into a Hitachi Limited L-8900 Automatic Amino Acid Analyzer for analysis. Norleucine was used for the internal standard.

### Trace elements

Unmated queens and unfertilized eggs from FF colonies and mated queens and fertilized eggs from FM colonies were sampled for trace elements. Each sample was placed in an acid-washed test tube with 0.5 ml of trace metal-grade concentrated nitric acid (HNO_3_, Thermo Fisher Scientific) and placed on a block heater. Each sample was digested by allowing the nitric acid to boil for approximately 30 min. After boiling, 0.2 ml of a 10 ppm yttrium solution (spectrum plasma emission standard grade), which was used as an internal standard, and 1.3 ml of deionized water (Millipore Synergy 185) were added to make a final solution volume of approximately 2.0 ml. Any undigested lipids were separated from the sample solution by adding 1.5 ml of high performance liquid chromatography (HPLC) grade chloroform (Thermo Fisher Scientific). The aqueous layer of each sample was measured using ICP-OES (PerkinElmer Optima 8000 DV) to determine the micronutrient content of each sample. Emission for each ion was detected at the following wavelengths: Ca, 317.933 nm; Cu, 324.754 nm; Fe, 238.204 nm; K, 766.491 nm; Mg, 279.079 nm; Mn, 257.610 nm; Na, 589.592 nm; Y, 371.029 nm; and Zn, 213.856 nm. Concentrations were determined using the calibration curve method after normalizing the emission intensity of each metal to that of scandium from the same sample. Standard solutions (0.1-10 ppm) each containing 1 ppm Y were made from a stock solution (Spex Certiprep Instrument Calibration Standard 2) containing 100 ppm Ca, Cu, Fe, K, Mg, Mn, Na, and Zn. Concentration measurements for all eight elements were obtained for each sample (Judd et al., 2010).

### Nutrient content

Unmated queens and unfertilized eggs from FF colonies and mated queens and fertilized eggs from FM colonies were sampled. Samples were homogenized in ice-cold buffer (0.1 mol/l Na_2_HPO_4_-KH_2_PO_4_, 0.1% Triton X-100, pH 7.4) in a proportion of 0.1 g of body weight to 1 ml of buffer. The homogenates were centrifuged at 3500 r/min (1191 ***g***) for 10 min, and the supernatant was used for analysis (Li et al., 2015). Protein, glucose, triglyceride and total cholesterol content was determined using commercially available spectrophotometric assay kits (Nanjing Jiancheng Bioengineering Institute, Jiangsu, China) following the manufacturer's protocols with some modifications. According to the Bradford (1976) method, protein concentrations were measured using bovine serum albumin as the standard. Glucose content of the supernatant was decomposed into gluconic acid and H_2_O_2_ by catalysis with glucose oxidase. Quinones were synthesized by the oxidative coupling polymerization reaction of 4-aminoantipyrene and phenol. The formation of the quinone conjugate was monitored via the variance in absorbance at 505 nm. The triglyceride and total cholesterol content was measured with an enzymatic cycling method on a microplate spectrophotometer (xMark, Bio-Rad). The determination parameters were as follows: 2-point rate assay, 2-point linear calibration, 37°C, and 500 nm main wavelengths. The secondary wavelengths of the triglyceride and the total cholesterol were 600 nm and 660 nm, respectively.

### Hormones and neurotransmitters

Unmated queens from FF colonies and mated queens from FM colonies were sampled. The purity of the two hormones JH III (Santa Cruz Biotechnology) and ecdysone (Selleckchem) was >65% and 99.25%, respectively. The purity of the three neurotransmitters serotonin hydrochloride (Sigma-Aldrich), octopamine hydrochloride (Sigma-Aldrich) and dopamine hydrochloride (Sigma-Aldrich) was >98%, >95% and >99%, respectively. The multiple reaction monitoring (MRM) experiment was performed according to the experimental procedure reported by Hikiba et al. (2013). Briefly, standard hormones and neurotransmitters were diluted in methanol across a range of concentrations (2.5 ppb, 25 ppb, 250 ppb, and 2500 ppb) to generate the calibration curves and for method validation. Calibration curves were generated using the same standard concentrations: 0.2, 1, 10, 50, 250, and 500 ng/ml. Peak areas were plotted and analyzed by linear least squares regression with weights of 1/x using the Analyst Software version 1.5 (AB SCIEX), and the experimental precision was indicated by the coefficient of variation (% CV). Termite samples that were stored at −80°C were sonicated at −20°C for 30 min and then at 4°C for 30 min. The chromatographic separation was performed on a Kinetex C18 HPLC (150×3.0 mm, 2.6 µm, 100 A). The column oven temperature was 40°C and the injection volume was 1 μl for all of the samples. Gradient elution was performed with 0.1% methanol (mobile phase A) at an initial flow rate of 0.6 ml/min. The levels of two hormones and three neurotransmitters in the samples were determined with QTRAP 5500 (AB SCIEX) in MRM mode.

### Reproductive genes

Unmated queens and unfertilized eggs from FF colonies and mated queens and fertilized eggs from FM colonies were sampled to assess the expression of three reproductive genes [*Vitellogenin 1* (*vtg 1*), *rab 11* and juvenile hormone esterase-like protein Est1 (*JHE 1*)] using qPCR. The termite samples (two termites) were pooled and crushed in 1.5 ml centrifuge tubes with liquid nitrogen using sterilized disposable tissue grinding pestles. The total RNA of the samples was extracted using RNAiso Plus (TaKaRa, Dalian, China) according to manufacturer's protocol. The purity and concentration of the extracted RNA were determined with a Thermo NanoDrop 2000 spectrophotometer. Approximately 1 μg RNA was converted to cDNA using PrimeScript™ RT reagent Kit with gDNA Eraser (Perfect Real Time) (TakaRa, Dalian, China). The cDNA products were then diluted with deionized water 20-fold as a template for the qPCR. The quantitative reaction was performed on a My IQ™ 2 Two-colour Real-time PCR Detection System (Bio-Rad, USA). Reaction mixtures, containing 5 μl of SYBR Premix Ex Taq™ II (TaKaRa, Dalian, China), 0.5 μl of forward primer (10 μM), 0.5 μl of reverse primer (10 μM), 2 μl of deionized water and 2 μl of template cDNA were performed under the following reaction conditions: 95°C for 30 s, followed by 40 cycles of 95°C for 5 s and 58°C for 30 s.

Based on the sequences of other termites, primers were designed using Primer Premier 5 (PREMIER Biosoft International, CA, USA) (*Vitellogenin 1*: AB520715.1, *Reticulitermes speratus*, forward, 5′-CAATCTCCTTAGTGCTTGCCGTA-3′, reverse, 5′-GTGACATAAGGGAAAGCCAAGTG-3′; *rab 11*: Unigene13177, *Odontotermes formosanus*, forward, 5′-TTGTGATGGAAGGGAAATGTATG-3′, reverse, 5′-TTATCTGAAGAGCACTGGAACGG-3′; *JHE 1*: GQ180944.1, *Reticulitermes flavipes*, forward, 5′-GCGTTAGAGAGGTAATGGCAAAA-3′, reverse, 5′-GGTAGAAGAATAAGGGAGCGGAG-3′; *β-actin*: DQ206832.1, *Reticulitermes flavipes*, forward, 5′-CTCAGGTGATGGTGTCTC-3′, reverse, 5′-CAGGTAGTCGGTCAAGTC-3′). Based on the acquired sequences of *R. chinensis*, specific primers for qPCR were designed by Beacon Designer 7.7 (PREMIER Biosoft International) (*Vitellogenin 1*: forward, 5′-AGGGAAAGCCAAGTGACGAA-3′, reverse, 5′-AAGCCAACGTACTCGACCTG-3′; *rab 11*: forward, 5′-GTAGTGCGTCAGGAGATCGT-3′, reverse, 5′-CTGAAGAGCACTGGAACGGA-3′; *JHE 1*: forward, 5′-AGCCAGTTTCACAGGAGACG-3′, reverse, 5′-AATAAGGGAGCGGAGGATCG-3′; *β-actin*: forward, 5′-CTCAGGTGATGGTGTCTC-3′, reverse, 5′-CAGGTAGTCGGTCAAGTC-3′). The gene expression of unmated queens and unfertilized eggs from FF colonies was calibrated with that of mated queens and fertilized eggs from FM colonies, respectively. The relative gene expression was calculated using the 2^−ΔΔ^Ct method (Livak and Schmittgen, 2001). The real-time PCR was performed on three biological replicates each containing three technical replicates.

### Statistical analysis

All statistical analyses were carried out in IBM SPSS Statistical 18.0 (SPSS Inc., Chicago, Illinois, USA). The results in the graphs represent the mean values ±s.e.m. Offspring number of female-female and female-male colonies in each stage were compared with each other using paired *t*-test. We applied one-way ANOVA to compare the egg volume between unfertilized and fertilized eggs at five developmental stages. Significant differences were analyzed using Tukey's multiple range test. The Student's *t*-test was used to compare the egg volume between unfertilized and fertilized eggs. The paired *t*-test was used to compare amino acids, trace elements, nutrients, hormones, neurotransmitters levels and reproductive genes expression between unfertilized eggs/unmated queens and fertilized eggs/mated queens.
